# Recovery of frog and lizard communities following primary habitat alteration in Mizoram, Northeast India

**DOI:** 10.1186/1472-6785-4-10

**Published:** 2004-08-06

**Authors:** Samraat S Pawar, Gopal S Rawat, Binod C Choudhury

**Affiliations:** 1 Wildlife Institute of India, Chandrabani, Dehradun 248 001, India; 2 Section of Integrative Biology, University of Texas, Austin, TX 78712, USA

## Abstract

**Background:**

Community recovery following primary habitat alteration can provide tests for various hypotheses in ecology and conservation biology. Prominent among these are questions related to the manner and rate of community assembly after habitat perturbation. Here we use space-for-time substitution to analyse frog and lizard community assembly along two gradients of habitat recovery following slash and burn agriculture (*jhum*) in Mizoram, Northeast India. One recovery gradient undergoes natural succession to mature tropical rainforest, while the other involves plantation of *jhum *fallows with teak *Tectona grandis *monoculture.

**Results:**

Frog and lizard communities accumulated species steadily during natural succession, attaining characteristics similar to those from mature forest after 30 years of regeneration. Lizards showed higher turnover and lower augmentation of species relative to frogs. Niche based classification identified a number of guilds, some of which contained both frogs and lizards. Successional change in species richness was due to increase in the number of guilds as well as the number of species per guild. Phylogenetic structure increased with succession for some guilds. Communities along the teak plantation gradient on the other hand, did not show any sign of change with chronosere age. Factor analysis revealed sets of habitat variables that independently determined changes in community and guild composition during habitat recovery.

**Conclusions:**

The timescale of frog and lizard community recovery was comparable with that reported by previous studies on different faunal groups in other tropical regions. Both communities converged on primary habitat attributes during natural vegetation succession, the recovery being driven by deterministic, nonlinear changes in habitat characteristics. On the other hand, very little faunal recovery was seen even in relatively old teak plantation. In general, tree monocultures are unlikely to support recovery of natural forest communities and the combined effect of shortened *jhum *cultivation cycles and plantation forestry could result in landscapes without mature forest. Lack of source pools of genetic diversity will then lead to altered vegetation succession and faunal community reassembly. It is therefore important that the value of habitat mosaics containing even patches of primary forest and successional secondary habitats be taken into account.

## Background

Evaluation of the importance of various processes determining community structure and function is an important topic in ecology. Unlike just a decade or so ago, few studies today question whether or not community assembly is strictly random, recognizing the role of both stochastic and deterministic processes [1]. This change can be attributed to accumulating data on organisation in experimental and natural communities, and new perspectives gained from the fields of evolutionary population ecology and phylogenetics ([2] e.g., [3,4]). It is worth noting that empirical tests for much of this theory have been attempted relatively recently in experimental microcosms research in particular, yielding valuable insights into the role of processes in driving long-term community dynamics [5].

This newfound view of community ecology is an excitingly realistic one, and has the potential to make valuable contributions to conservation biology as well [6,7]. However, though studies on long term dynamics of communities are obviously important, they are extremely difficult to implement. A vast majority of field studies are restricted to exploring correlates and predictors of species diversity and other emergent community properties. This is in part due to the problems associated with studying complex natural communities. But to a great extent, difficulties also arise because community ecology studies have traditionally been skewed towards relatively long-lived vertebrate groups, or are restricted to short study periods due to logistical constraints, especially in the tropics [8,9]. Although these studies yield valuable information, they are carried out at timescales that at best, give insight into short-term processes, providing limited information about community organization, persistence, or assembly.

Circumventing this problem is obviously very difficult. One possible approach is to study communities along gradients of habitat succession using space-for-time substitution (SFT) to obtain chronosequential communities [10]. Thus, instead of studying changes in a single community over time, successional habitats of known ages that can be arranged on a temporal gradient are compared. This method can reveal changes in community structure, environmental predictors of these changes, and provide estimates of the rate of community change [11-14]. Although many studies have examined recovery of faunal communities with tropical forest regeneration, a vast majority have been restricted to one or two vertebrate (birds, small mammals) and invertebrate (ants, beetles) groups [10]. For example, 19 of the 33 studies reviewed by Dunn [10], were on vertebrates, out of which only two were on amphibians and/or reptiles. Considering that amphibians and reptiles are ectothermic and have life history traits different from mammals and birds [9], more data on these taxonomic groups is important to test the generality of conclusions about effects of tropical habitat alteration on fauna.

This study takes an SFT approach to compare changes in frog and lizard community structure in two contrasting habitat succession gradients: (a) 1-yr *jhum *fallows giving way to mature forest, and (b) 1-yr *jhum *fallows planted over with teak, leading to monoculture stands. Slash-and-burn or shifting cultivation (*jhum*) agriculture involves clearing and burning of forest patches, so the original rainforest communities are effectively obliterated, and succession involves recovery of communities from scratch. The following questions were addressed in this study:

1. How much does frog and lizard community succession differ between the two gradients of habitat recovery?

2. Does composition of the entire community change in synchrony, or does the recovery pattern differ between subcommunities such as frogs vs. lizards and guilds?

3. What aspects of habitat change influence frog and lizard community recovery, and if habitat parameters are linked to niche axes, do they predict changes in guild composition?

4. Do successional changes in guilds also show trends in phylogenetic structure? This last question is expected to yield interesting insights into possible evolutionary mechanisms underlying changes in community composition [15], but has not explicitly been addressed in previous work on faunal recovery during tropical forest regeneration (cf. [10], and references therein).

In this paper, a chronosere is defined as a habitat that has recovered from perturbation for a known length of time, and can be assigned a place in the SFT. An assemblage is the set of all species of a taxonomic group in a landscape of interest. Ecological groups (EGs) are species' subsets of the assemblage with similar niche characteristics. Communities comprise species of the assemblage which share a habitat stratum (i.e., chronosere) in the landscape. Guilds are members of the EGs that actually coexist in the same chronosere i.e., belong to the same community, and are thus likely to have ecological and evolutionary interactions (cf. [16]).

To draw inferences about what aspects of habitat change determine sequential communities, habitat and frog-lizard community data were analysed hierarchically. As a first step, species richness and turnover of frog and lizard communities along habitat recovery gradients was summarised, and the entire assemblage classified into ecological groups (EGs) based on niche similarities. Guilds identified from this classification were then examined for phylogenetic structure. Using factor analysis, orthogonal combinations of variables that described biotic and abiotic aspects of habitat transition were extracted. We then tested for correspondence between these composite variables and composition of frogs and lizard communities and guilds. Based upon the relationships between different habitat factors and frog and lizard communities, variables were interpreted as composite adaptive zones, and we tested whether they predicted successional changes at different levels of community organization.

## Results and Discussion

### Gradients of vegetation recovery

Table 1 shows details of the chronosere sampling plots (see Additional file 2 for photographs of chronoseres). Both the 1-yr post-*jhum *fallows (plots jh1A and B) were dominated by herbaceous plants, tall grass, shrubs and wild bananas, along with saplings and surviving crop plants. The 4 to 5-yr post-*jhum *plot (jh5) was dominated by almost homogeneous stands of the bamboo *Melocanna baccifera*, interspersed with a few shrubs and trees. Herbs were rare, and the understorey sparse. The 7 to 10-yr post-*jhum *plot (jh10) was very similar to jh5. However, here the bamboo culms were more sparsely distributed, and along with *M. baccifera*, two other bamboos- *Dendrocalamus longispathus *and *Bambusa tulda *were in greater abundance, and woody plants were relatively more common. Compared to the other plots, a larger area was included in the 30 to 35-yr *jhum *plot (jh35) because it contained a greater range of ages and hence perhaps more variability. Also, this was the only accessible site in the study area that represented a chronosere aged between 30–50 years. Although *M. baccifera *was common, this site had a greater abundance of other bamboos and trees than any of the previous stages. Though most trees were small, woody vegetation formed a significant part of the canopy. Herbs and shrubs were rare, and the understorey generally sparse.

**Table 1:** Details of sampling plots. Plot ages were determined by consultation with local people. The labels in the first column are used to identify plots throughout the rest of the paper.

**Plot**	**Details**	**Size (ha.)**
**jh1A**	1 year *jhum *fallows, cultivated and abandoned in 1998	3–4
**jh1B**	1 year *jhum *fallows, cultivated and abandoned in 1998	3–4
**jh5**	Two adjoining, indistinguishable 4–5 year *jhum *fields cultivated and abandoned in 1994 & 1996 respectively	4–6
**jh10**	Three adjoining, indistinguishable 7–10 year *jhum *fields cultivated and abandoned between 1988 & 1991	4–6
**jh35**	Five adjoining, indistinguishable 30–35 year post-*jhum *fields cultivated and abandoned between 1963 & 1969	8–10
**tk4**	4 year old teak plantation, planted in 1994	3–4
**tk 22**	Subset of a 22 year old teak plantation, planted in 1977	4–6
**matA**	Subset of slightly disturbed contiguous mature forest	4–6
**matB**	Subset of undisturbed contiguous mature forest	4–6
**matC**	Subset of undisturbed contiguous mature forest	4–6

The three mature forest plots (matA, B, and C) were of untraceable age (probably >100 years old; [12]). They were characterized by high tree density, canopy cover, and a sparse understorey with few herbs or true shrubs (not tree saplings). One of the sites (matA) was slightly disturbed by dead wood and palm leaf extraction, and had a relatively dense understorey in places. Bamboos were mainly restricted to moist gullies, and occasionally in the understorey.

The 4-yr teak plot (tk4) was a young plantation characterized by a monodominant stand of teak trees. The understorey was sparse, with some tall grass, shrubs (mainly *Lantana camara*) and occasional herbs. The 22-yr teak plantation site (tk22) had a monotonous, uniform structure characteristic of a mature, managed teak monoculture. Undergrowth was sparse, consisting mostly of tall grass and *Lantana *sp. Table 2 summarises differences in four habitat parameters that show broad contrasts between chronoseres. These results are very similar to those of a another study in the same region [12]. For the purposes of these comparisons, data for the two undisturbed mature forest plots matB and matC are presented together because they are were very similar in macrohabitat characteristics.

**Table 2:** Differences in four macro-habitat parameters across plots. All variables were normally distributed, but not homoscedastic (Levene's test, p < 0.001), so Tamhane's T2 (a conservative pair wise comparisons test based on a t test) was used as a post-hoc multiple range test (F ⇒ F-ratio of one-way parametric ANOVA; ** ⇒ p < .005; * ⇒ p < .05; – ⇒ Not significant;).

**(a) Tree density (F = 79.232)**								
Plot	Mean / 250 m^2 ^± S.E.	jh1A&B	jh5	jh10	tk4	tk22	jh35	matA

jh1A&B	00.41 ± 0.26							
jh5	01.52 ± 0.96	**-**						
jh10	03.38 ± 0.95	**-**	**-**					
tk4	34.05 ± 3.04	**	**	**				
tk22	24.45 ± 0.87	**	**	**	**			
jh35	15.32 ± 1.30	**	**	**	**	**		
matA	27.67 ± 2.05	**	**	**	**-**	**-**	**	
matB+C	20.33 ± 1.76	**	**	**	**	**-**	**-**	**-**

**(c) Bamboo culm density (F = 194.30)**								

Plot	Mean / 25 m^2 ^± S.E.	jh1A&B	jh5	jh10	tk4	tk22	jh35	matA

jh1A&B	00.00							
jh5	96.36 ± 5.05	**						
jh10	62.86 ± 4.85	**	**					
tk4	00.00	**-**	**	**				
tk22	00.00	**-**	**	**	**-**			
jh35	30.26 ± 3.85	**	**	**	**	**		
matA	00.01 ± 0.21	**-**	**	**	**-**	**-**	**	
matB+C	01.14 ± 0.15	**-**	**	**	**-**	**-**	**	**-**

**(b) Canopy cover (F = 139.38)**								

Plot	Mean (%) ± S.E.	jh1A&B	jh5	jh10	tk4	tk22	jh35	matA

jh1A&B	12.07 ± 1.67							
jh5	67.91 ± 2.79	**						
jh10	69.79 ± 1.71	**	**-**					
tk4	37.25 ± 2.58	**	**	**				
tk22	51.33 ± 4.61	**	**	**	**			
jh35	76.70 ± 1.62	**	**-**	**-**	**	**		
matA	72.64 ± 2.03	**	**-**	**-**	**	**	**-**	
matB+C	80.68 ± 1.86	**	**	*	**	**	**-**	**-**

**(d) Shrub density (F = 12.21)**								

Plot	Mean / 25 m^2 ^± S.E.	jh1A&B	jh5	jh10	tk4	tk22	jh35	matA

jh1A&B	25.85 ± 2.86							
jh5	25.59 ± 3.31	**-**						
jh10	19.58 ± 2.29	**-**	**-**					
tk4	11.28 ± 1.19	**	**-**	**-**				
tk22	13.99 ± 2.08	**	**-**	**-**	**-**			
jh35	27.58 ± 3.21	**-**	**-**	**-**	*	**-**		
matA	45.24 ± 4.26	**	**	**	**	**	*	
matB+C	39.37 ± 4.04	*	**-**	**	**	**	**-**	**-**

**Figure 1:**
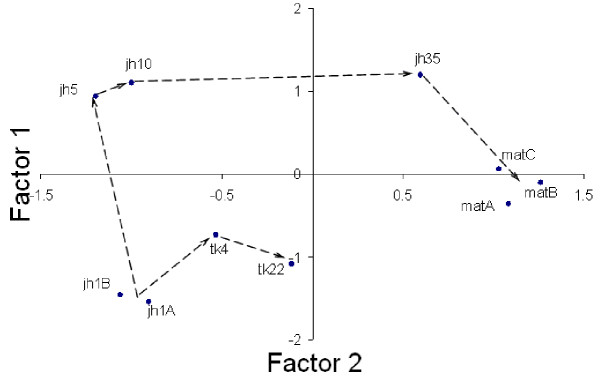
Plot of scores of first two PCA factors. Vectors are drawn to show the trajectories of the two gradients of habitat recovery. The chronosere codes are as follows- *jh1A*, *1B*, *5*, *10 *&*35*: *jhum *fallows ranging from 1 to 35 years; *tk4 *&*22*: teak plantation 4 and 22 years old; *matA*, *B*, &*C*: mature forest plots. See methods and Table 1 for more details.

Eight factors were extracted after Principal Component Analysis (PCA), and by Varimax rotation of the factor structure, which explained a cumulative 85.8% of the variation (see methods). Eigenvalues, factor loadings and factor scores are given in Additional file 3. The ordination of the sampling plots based on scores of the first two PCA factors is shown in Figure 1. This ordination was very similar to one obtained by non-metric multidimensional scaling (NMDS) [17]. The two gradients of vegetation recovery have very different trajectories of change in habitat attributes. The predominant macro-habitat characteristic along the factor 1 axis is high bamboo abundance, while high positive loading on the factor 2 axis indicates tree-forest dominated habitat. The gradient towards mature forest succession includes intermediate stages dominated by bamboo, which are succeeded by a tree dominated forest.

In general, although there is a change towards a tree dominated habitat in both recovery gradients, the end result is very different because the 22 year teak plantation is a monoculture, whereas the mature forest consists of a diverse tree community.

### Successional changes in frog and lizard communities

**Figure 2:**
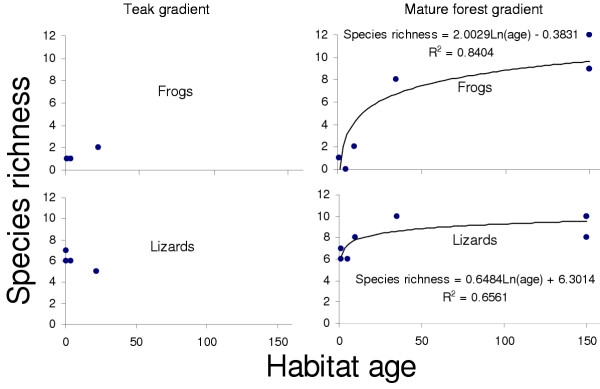
Change in species richness of frogs and lizards with chronosere age along teak plantation and mature forest recovery gradients. Both gradients have 1 yr *jhum *fallows (jh1A&B) as the starting point. The number of species increases logarithmically with succession towards mature forest for both taxonomic groups, but the change is much more striking in the case of frogs. Species richness does not seem to change much with recovery time on the teak gradient. The age of mature forest, known to be >100 years old, was assigned an arbitrary value of 150 years.

**Figure 3:**
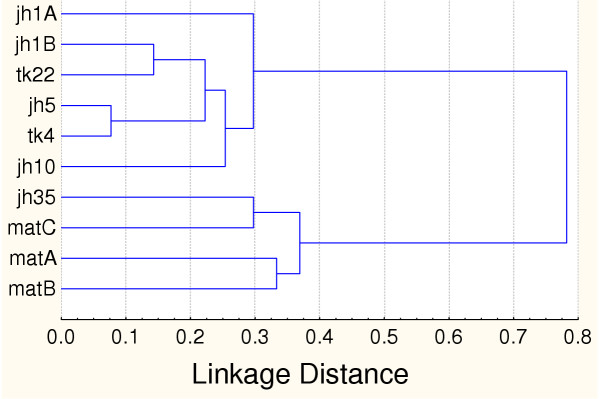
Dendrogram of similarities in frog and lizard (combined) communities across plots. See text for explanation. Community overlap was calculated with the Bray-Curtis measure, and sites clustered using the UPGMA algorithm.

The three sampling techniques used in conjunction during the study (see methods) yielded sixteen frog and seventeen lizard species. Figure 2 shows changes in species richness, and Figure 3 differences in community composition for frogs and lizards along the two recovery gradients. Clearly, there are distinct similarities in overall community composition between the early *jhum *fallows and teak plantation communities on one hand, and the mature forest and the 35 year *jhum *fallows on the other.

The pattern of recovery is very different for the two gradients. For the mature forest succession gradient, the rate of frog and lizard community recovery is similar to that found for birds by Raman [12] in the same region in Northeast India, with the community approaching mature forest characteristics after about 30 years. Even more remarkably, this timescale is also comparable with those reported by similar studies on other fauna elsewhere in the tropics [10]. The gradient from *jhum *to mature teak plantations on the other hand, seems to show little change in species richness or composition even after 22 years of plantation growth.

It is worth noting that there are dissimilarities in the manner of species accumulation for frogs vs. lizards. There is much less augmentation of species number in the case of the latter, the main reason for this being that younger chronoseres support more lizard than frog species richness. Species accumulation curves (see Additional file 4) show that these differences are not sampling artefacts. Moreover, across chronosere species turnover (see methods) for lizards is significantly higher than that for frogs (Student's t-test, two tail p < 0.05), indicating that lizard community succession was characterized by relatively high replacement and low accumulation of species.

#### Guilds

**Figure 4:**
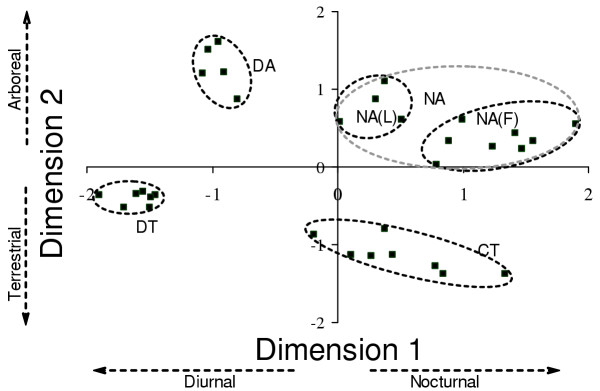
NMDS configuration showing ecological groups (EGs) of frogs and lizards in the assemblage. Each point represents a species. For this configuration, stress = 0.14712 and RSQ =.90076 [17]. Broad characteristics of EGs are as follows: DA= Diurnal, arboreal; NA(L)= Nocturnal, arboreal, all lizards; NA(F)= Nocturnal, arboreal, all frogs; CT= Crepuscular-nocturnal, terrestrial; DT= Diurnal, terrestrial. See Additional file 4 for EG species' identities.

Ecological groups defined by non-metric multidimensional scaling (NMDS) of the niche based dissimilarity matrix for the entire assemblage are shown in Figure 4. The NMDS configuration was derived in 2 dimensions with low stress and high RSQ values, indicating a very good representation of actual niche dissimilarities [18]. On dimension 1, the dominant niche characteristic determining high negative loadings is arboreality, and high positive values indicate that the species is predominantly terrestrial. On dimension 2, higher positive values indicate predominantly diurnal diel activity pattern, and negative values indicate crepuscular and/or nocturnal activity pattern. Identities of species in each group are in Additional file 4. Of the five EGs, two consist of both frogs and lizards: the nocturnal arboreal (NA) group with eight species of frogs (mostly tree frogs) and four species of lizards (all gekkonid lizards), and the crepuscular-nocturnal terrestrial (CT) group, which consists of seven frogs and one crepuscular-nocturnal lizard. The diurnal arboreal group (DA) consists of five agamid lizards, some of whom are occasionally terrestrial. The diurnal terrestrial (DT) group consists of six skinks and one lacertid lizard. More detailed natural history descriptions of these species can be found in Pawar [17].

**Figure 5:**
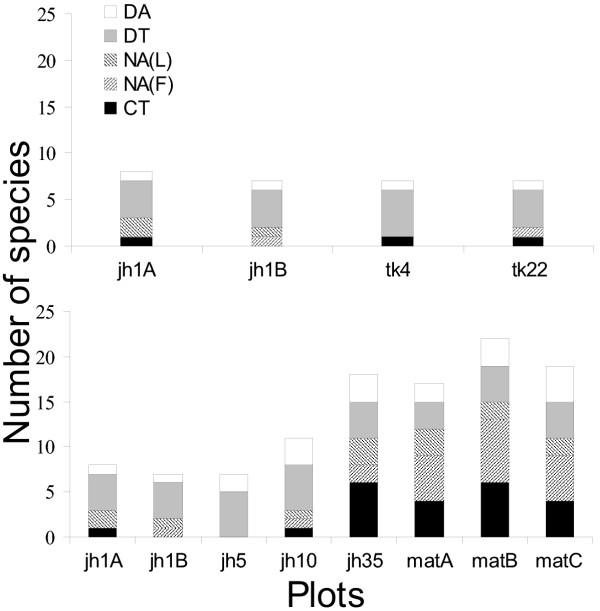
Representation of ecological groups along gradients of habitat recovery. Each EG for a particular habitat is effectively a guild. Note that the number of guilds increases with succession along the *jhum *to mature forest gradient, but not along the teak gradient. See Figure 4 for guild identities, and Additional file 4 for species' that make up each EG.

Figure 5 shows how EGs are represented along the two recovery gradients. In this paper, representatives of each EG in a sampling plot are considered guilds of that chronosere. Clearly, the number of guilds as well as number of species per guild increases with succession along the gradient leading to mature forest, but not along the one leading to teak monoculture. The species accumulation during natural forest succession is mainly due to augmentation of crepuscular and nocturnal guilds. It is also worth noting that the DT and DA groups, which are consistently present along both gradients of recovery, also have the maximum niche overlap (distance between pairs of species is the smallest for these groups in the NMDS niche space). The implication of this fact is discussed below. It is due to these two guilds that successional lizard communities show the high species turnover and low accumulation noted above.

#### Phylogenetic structure

**Figure 6:**
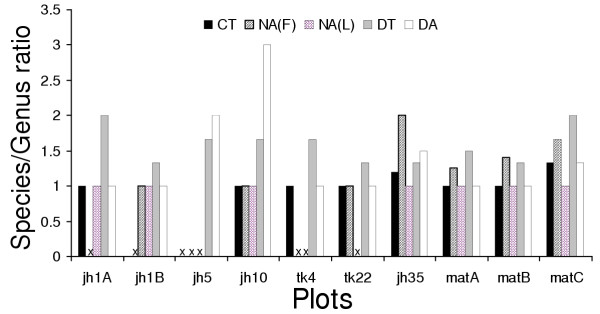
Trends in phylogenetic structure (species/genus ratio) in five guilds across chronoseres. An "x" indicates that a guild is absent. The S/G ratio increases with time of habitat recovery in the nocturnal guilds, but not for the diurnal guilds. See text for discussion.

Figure 6 shows the ratio of species to genera (S/G ratio) of guilds in different chronoseres. The S/G ratio increased with succession towards mature forest in the NA guild, and to a lesser extent, in the CT guild. The S/G ratio of the NA, DT and DA guilds was variable, and did not change directionally with habitat recovery. In general, across all chronoseres irrespective of which recovery gradient they belonged to, the number of guilds represented in each chronosere was positively correlated with phylogenetic structure (S/G ratio averaged across guilds; Spearman R = 0.92, p < 0.0002) and the species richness of the chronosere (R = 0.88, p < 0.01). This suggests that the ability of chronoseres to support a larger number of guilds predicts species number as well as phylogenetic structure.

These results on successional changes in guild structure and representation indicate a distinctly non-random sequence of community assembly, as certain guilds appear in later stages, followed by increase in their species richness and in many cases, phylogenetic structure. Habitat attributes that determine these changes are explored in the next section (see below).

### Correspondence between habitat factors and frog-lizard community succession

Table 3 shows the results of correspondence tests between Euclidean dissimilarity matrices calculated from the eight PCA factors and frog-lizard species compositional dissimilarity matrices for different levels of community structure. As the factor structure of the PCA analysis was rotated to maximize the orthogonality of factor loadings, these matrix correspondence tests are statistically similar to performing partial mantel tests (partial correlation) with multiple variables [19]. The hierarchical nature of the correlations in Table 3 and the fact that guilds are correlated with different, orthogonal composite variables is interesting, and offers answers to the third question addressed this paper: what aspects of habitat change influence frog and lizard community recovery at different levels of community organization?

**Table 3:** Correlation between dissimilarity matrices based on eight PCA factors (unsquared Euclidean distances), and different levels of community organisation (Jaccard's index). The correlation coefficients are followed by significance (p) values in parentheses. The p-values were estimated by 1000 Monte Carlo randomizations of each pair of matrices. Correlations with p values >0.05 not reported. See the text and Additional file 3 to see loadings of habitat variables for the PCA factors.

**Community/guild**	**Factor 1**	**Factor 2**	**Factor 3**	**Factor 4**	**Factor 5**	**Factor 6**	**Factor 7**	**Factor 8**
**Frogs + Lizards**		0.762 (0.003)						
**Frogs**		0.5031 (0.003)					0.467 (0.019)	
**Lizards**		0.672 (0.005)						0.375 (0.033)
**CT**	0.331 (0.035)	0.417 (0.016)		0.402 (0.016)				
**DA**		0.630 (0.008)						
**DT**		0.608 (0.003)						
**NA(F)**		0.347 (0.025)					0.482 (0.037)	
**NA(L)**		0.362 (0.004)		0.417 (0.030)				

#### Higher-order community structure

The strongest association is between factor 2 and overall species composition (frogs and lizards combined) across chronoseres. Factor 2 was strongly and non-linearly correlated with age along both teak and mature forest succession gradients (logarithmic fit, R^2 ^= 0.85, and 0.97, respectively), and represents deterministic, linear aspects of vegetation succession. It has high positive loadings for tree species richness, and macro-habitat variables such as tree density, canopy cover, and canopy height, most of which increase deterministically along both gradients of habitat change. Among the measured variables, these are primary and independent, which over time drive changes in secondary (microhabitat) variables such as bamboo density, shrub abundance, and various measures of habitat heterogeneity (see methods). This factor clearly influences species composition at all levels of frog and lizard community structure.

#### Frog vs. Lizards

Along with factor 2, the frog subcommunity was also associated with factor 7, which shows no significant age determinacy along either gradient of habitat recovery. This factor has high positive loading for soil moisture content, which is an important limiting factor for frogs. The lizard subcommunity was associated with factor 8 along with factor 2. Factor 8 has high loading for soil moisture variability, which is highest in chronoseres with spatial variation in insolation. This factor is crucial for diurnal lizards, many of which are heliotherms. Factors 7 and 8 are probably also surrogates of unmeasured or unclassified variables which influence successional changes in these two communities.

#### Guilds

Three out of five guilds are secondarily correlated with factors orthogonal to factor 2. The two that were not, i.e., the diurnal-arboreal (DA) and diurnal-terrestrial (DT) groups, were correlated with factor 2. This suggests that in contrast to other guilds, these two, which are both made up only of lizards, are directly influenced by a hierarchically higher order of habitat attributes.

These were also the two groups that showed non directional trends in species richness as well as phylogenetic trends along habitat recovery gradients (Figures 5 and 6).

Along with factor 2, the crepuscular-nocturnal terrestrial (CT) group was correlated with factor 1 and 4. Factor 1 scores increase and then decrease with plot age along both habitat recovery gradients (2^nd ^order polynomial fit, R^2^= 0.99, and 0.82, respectively). This factor had high loading (defined as ≥ ± 0.65; see Additional file 3) positive for both macro- and micro-habitat variables such as bamboo density canopy cover, leaf litter cover and depth and negative loadings for many habitat heterogeneity variables such as CVs of canopy cover and litter cover. These variables are interpretable as ones that are associated with, or influence ground microhabitat conditions. Factor 4 decreased logarithmically with age along both teak and mature forest gradients (R^2 ^= 0.62, and 0.34, respectively). This factor had no strong loadings, but is associated with shrub density, canopy height variability and tree density, all of which also affect ground cover, and can be considered macrohabitat variables.

The nocturnal arboreal frog group (NA(F)), was correlated with factor 7, which is non-deterministic with respect to chronosere age. This factor as a high loading for soil moisture, which by itself is difficult to interpret as a variable directly affecting this ecomorphological group. It is likely that this factor is a surrogate for an unmeasured or unclassified variable. Lastly, the nocturnal arboreal lizard group (NA(L)) is correlated with factor 8 along with factor 2. Factor 8 shows a weak negative linear relationship with recovery age along both gradients gradient (R^2 ^= 0.209 and 0.18 for teak and mature forest gradients, respectively). It has high positive loading for CV of soil moisture, which as mentioned above, is highest in chronoseres with spatial and/or temporal variation in insolation. Among the measured variables, factor 2 probably subsumes most habitat parameters that affect both NA groups directly (see next section).

### Adaptive zones?

**Figure 7:**
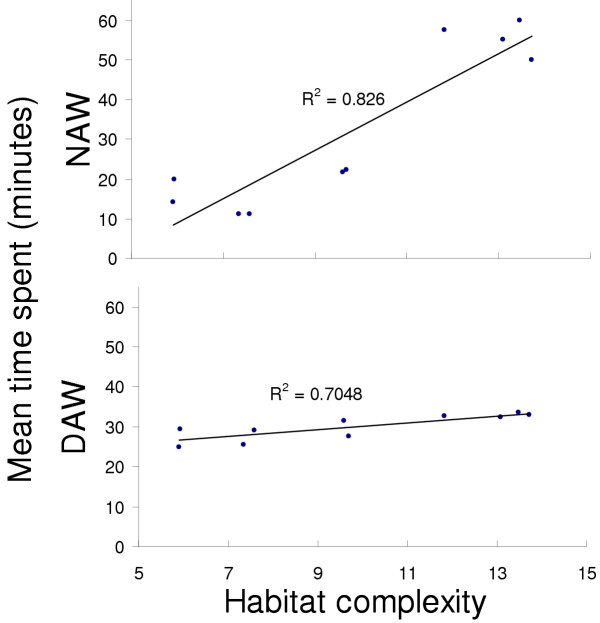
The relationship between transect sampling time and habitat complexity. The NAW time varies across habitat, and increases with habitat complexity, whereas DAW is constant. The reason for this is that more complex habitats needed more searching time. The index of complexity was calculated by summing the coefficients of variance for various understorey habitat structure variables. Sample sizes of transects were: jh1A = 15, jh1B = 13, jh5 = 14, jh10 = 16, tk4 = 15, tk22 = 15, jh35 = 38, matA = 17, matB = 28, matC = 21.

Can biologically meaningful adaptive zones be interpreted from these associations? As each factor is orthogonal with respect to the others, factors subsume different habitat variables, or their variability in the same variable. Note that the composite variable represented by each factor consists of negative as well as positive loadings of variables. This means that if a guild was associated with a factor, both positive and negative trends in different variables affected it simultaneously, together representing a composite adaptive zone. However, an important fact to consider here is that these "adaptive zones" thus identified may actually be surrogates for actual sets of unmeasured variables. Raman [12] inferred that floristics (tree species composition) and physiognomy (vertical stratification) were the dominant habitat attributes that independently predicted changes in bird species composition at the level of communities, but not at much at the level of guilds. In the case of frogs and lizards, factor 2, which includes a measure of floristic attributes (tree species diversity), is a strong predictor of frog and lizard community composition at all levels. But factor 2 also includes numerous structural attributes that are correlated with tree species diversity, from canopy height to understorey and ground habitat structure, all of which have equal or higher positive loadings. Also, Figure 7 shows that understorey habitat complexity increases with post-*jhum *succession towards mature forest.

Thus, it is difficult to infer the extent to which tree species diversity *per se *influences frog and lizard community structure. Previous work has shown that unlike endothermic vertebrates, amphibian and reptile distributions are likely to be influenced more strongly by abiotic rather than biotic features [20]. The effect of physiognomy on the other hand, is definitely important, though at a different scale than for birds. The idea that a habitat with higher structural complexity will support more species [21-25], and have a strong influence on re-colonisation success [26], has been shown for amphibians and reptiles (but see [27]). It can therefore be inferred that factor 2 subsumes nested subsets of biotic and abiotic variables that directly affect the (mean) fitnesses of species' populations in different guilds. Also, it is clear that guilds are also associated with other, independent variables sets that can be considered to comprise additional aspects of each member species' adaptive zone.

Factors 3, 5, and 6 showed no significant association with any level of community composition. The obvious reason for this appears to be that unlike other factors, these are completely non-deterministic with respect to age of succession, thus representing temporally and/or spatially stochastic attributes that were unlikely to show any influence on the conspicuously deterministic nature of frog and lizard community and guild (except for the DT and DA groups) succession (See Figures 2,3,5 and 6). At this resolution, it is impossible to say whether these variables are adaptively significant for certain subgroups/species of frogs and lizards or not. Nevertheless, this complex, nested pattern of these alleged adaptive zones, is ecologically realistic (see [28]). Although difficult to interpret at this level of resolution, this hierarchical partitioning of variables is an indicator of which attributes of habitat change influence community assembly and turnover with such gradients of vegetation succession.

### Composite variables and successional changes in community characteristics

Table 4 shows the predictive ability of the different habitat factors for species richness, guild abundance, and phylogenetic structure in communities and guilds. As expected, factor 2 predicts increase in overall species richness, number of guilds represented, and number of species per guild. However, it does not predict overall phylogenetic structure (measured as the average of S/G ratios across guilds represented in each chronosere). Instead, it predicts the phylogenetic structure of all guilds except DT. Factor 1 predicts species richness as well as S/G ratio in the DA group, and factor 4 predicts the S/G ratio of NA(L). No community characteristics were correlated with factors 3,5,6,7 or 8.

**Table 4:** Correlation between availability of composite variables (factor scores) and various measures of community change. Coefficients are Spearman's R, with p-levels in parentheses. Correlations with p > 0.05 not reported. No community characteristics were correlated with Factors 3,5,6,7 or 8.

	**Factor 1**	**Factor 2**	**Factor 4**
**Species Richness**			
Overall		0.77 (0.009)	
DA	0.81 (0.004)		
DT			
CT		0.91 (0.000)	
NA(F)		0.82 (0.003)	
NA(L)			
**Number of Egs**		0.69 (0.028)	
**Species per EG**			
Overall		0.72 (0.019)	
CT			
DA			
DT			
NA(F)			
NA(L)			
**S/G ratio**			
Averaged across EGs			
CT		0.66 (0.036)	
DA	0.85 (0.002)		
DT			
NA(F)		0.71 (0.020)	
NA(L)			0.80 (0.006)

The DT group does not show correlation with any of the factors. Interestingly, this ecological group along with the DA group, also occupies the smallest niche space (having the maximum niche overlap in the NMDS space; see Figure 4), has the most consistent presence across chronoseres (but with species turnover) and a phylogenetic structure that varies non-directionally along successional gradients (Figure 6). Similar patterns have been observed for diurnal terrestrial herpetofauna (which are largely lizards) elsewhere [29]. Members of this group also have the highest population densities, and most have wide geographic distributions (Pawar, unpublished data). All these data strongly suggest that this guild is not resource constrained in chronoseres along the habitat recovery gradients, and is more randomly assembled during recovery than any of the other groups.

In general, these results help explain the trends seen in Figures 2,3,5 and 6 by indicating attributes of habitat change that drive changes in community structure in successional frog and lizard communities. The succession of *jhum *fallow towards mature forest involves a deterministic, directional change in attributes that allow coexistence of successively more speciose and phylogenetically structured communities. In terms of change in species richness, these results are qualitatively similar to those of similar work on bird, butterfly, and reptile communities [11-13]. Previous work has not however attempted to look at phylogenetic structure for such successional communities. The *jhum *to teak monoculture gradient also has many aspects of deterministic habitat change, but apparently not for the variables that are essential for a diverse community. The trajectory of habitat change also indicates that this pattern is unlikely to change with transition towards older plantations either. No previous data exists on herpetofaunal community changes in post-*jhum *monoculture plantations.

## Conclusions

By comparing disparate trajectories of habitat change and recovery of different taxonomic groups, this study provides useful insights into faunal community change in response to habitat recovery. To summarise, the results show that (1) The two gradients of habitat recovery are very different and accordingly affect frog and lizard community assembly differently, (2) Although both groups increased in species richness with habitat recovery, lizards had higher species turnover, combined with lower species augmentation within each recovery gradient (3) Looking at a finer scale of community organization, assembly appears to be driven by changes in guild representation and composition, where some guilds change directionally with age of habitat recovery by species augmentation, while others change by species turnover (4) Guilds that showed directional increase in species richness also increased in phylogenetic structure (5) Hierarchies of community organisation were affected by composite, nested habitat attributes that correspond to particular niche axes, and (6) the increase in species richness along the mature forest gradient in contrast to lack of change along the teak gradient was due to availability (or lack thereof) of variables that comprise these complex adaptive zones. Also, the results show that a niche-based guild classification reveals patterns that would have been hidden in the gross response pattern of the entire community.

Some indication of the qualitative nature of potential evolutionary and ecological processes in community turnover comes from the fact that changes in phylogenetic structure are tied to guild structure in the communities. Using phylogenetic techniques, recent work has demonstrated the importance of evolutionary adaptation in assembling ecological communities [4]. It is clear that specialisation on different subsets of resources, in a habitat drive the origin and as well as persistence of diversity [30]. Frogs and lizards have incongruent patterns of community succession, mainly because they generally differ in fundamental niche dimensions axes such as diel activity [31]. However, although most lizards are diurnal and most frogs nocturnal, there are many sub-lineages that are an exception, and species do share niche space transcending taxonomic boundaries (ecological groups in this paper). Such subgroups probably have congruent ecological and evolutionary dynamics.

It is an open question as to what extent vegetation succession leads to changes in the number of adaptive peaks and corresponding changes in mean fitnesses of species' populations such that multiple species can persist in the same habitat. In more ecological terms this is same as asking how habitat succession leads to changes in niche availability, occupancy, and overlap (due to character displacement, for example). Another related question, that was partly explored using the S/G ratios in this paper, is whether similar adaptive zones (or niches or adaptive peaks) tend to be occupied by more closely related taxa. The results here do indicate that this may be true for such gradients of community change, as phylogenetic and guild structure increase directionally and in tandem with succession towards mature forest. Whether this change is driven by immigration from the regional gene pool or due to local divergent adaptation is an interesting question [15]. Reptiles, and to a greater extent amphibians, have limited dispersal ability compared to most vertebrates. This distinction in itself may drive differences in local adaptation and community assembly from other biotic groups.

### Conservation issues

The time scale of recovery on the *jhum*-rainforest succession gradient, which is about 30 years for both frogs and lizards, and suggests that recovery of diverse communities can be relatively fast, as has been reported for other fauna [10]. However, this pattern of community recovery (or re-assembly) is tightly coupled to changes in certain sets of habitat attributes, which in turn are dependent upon vegetation succession wherein post-*jhum *chronoseres are gradually replaced by trees. This vegetation succession is obviously reliant on seed rain/dispersal from nearby mature forests. In this region and many other areas Southeast Asia, apart from continued pressure from shifting cultivation and shortening cultivation cycles, it has also become popular practice to plant and maintain monocultures of timber species. As the results of this study indicate, such plantations are unlikely to support natural recovery of faunal communities, and will harbour lower biological diversity compared to primary forest.

It is possible that the combined effects of short *jhum *cycles, plantation forestry and invasion by non-native species such as *Lantana *and *Eupatorium *will lead to the local extirpation of even remnant forest patches. This loss of recolonisation pools for flora and fauna, will alter natural trajectories of succession, and strongly impact the biological diversity supported by the landscape. It is therefore important that conservation and prioritisation agencies in these areas consider the value of habitat mosaics containing even small patches of primary forest vegetation.

## Methods

The study was carried out from November 1998 to April 1999 in and around Ngengpui Wildlife Sanctuary (WLS; 21°56'N – 24°31'N and 92°16'E – 93°26'E) in south Mizoram, Northeast India. The study area covers about 200 sq. km. (see Additional file 1 for maps). A combination of high annual precipitation and temperature, and low elevation supports a predominantly tropical evergreen [32] climax vegetation in the area. Shifting cultivation is the primary mode of agriculture here. While most of the area within Ngengpui WLS is mature or primary forest, the surrounding areas are a mosaic of bamboo-dominated sites, mature forest fragments, teak *Tectona grandis *plantations and abandoned shifting cultivation (*jhum*) fallows of varying ages. All primary forest is referred to as "mature forest" throughout the paper because it is often difficult to determine the age of ostensibly primary tropical forest, especially in areas with poorly known history of land use and recovery [12,33]. Further details and supporting literature about the geology, vegetation, and land use patterns in the study area can be found in Pawar [17].

### Sampling plots

Ten sampling plots representing mature and successional vegetation stages of known ages were established [17] (Table 1). To control for recolonisation potential, all secondary plots were selected such that at least 50% of the perimeter abutted mature forest, and the edge was within 100 m from contiguous mature forest. All plots had a slope of 0–20° and were within an altitudinal range of ca. 150–350 m above sea level. As the study was focused on terrestrial frogs and lizards, all plots were at least 100 m away from large perennial water bodies. To minimize spatial autocorrelation, all plots were at least 2 km (straight distance) from each other, with the replicates (e.g., the two 1 yr fallows) being the furthest apart (ca. 10 km).

### Vegetation sampling, habitat variables and gradients of habitat recovery

Vegetation composition and habitat structure variables were sampled on randomly located 10 × 25 m belt transects [13,17]. Transects were cut short whenever an edge of the site was reached. The number of transects sampled were, six each in Jh1A, Jh1B, Jh5, Jh10, and Jh35, and five each in tk4, tk22, matA, matB and matC. Tree density and tree species richness was sampled on the whole area of each transect. All trees >20 cm GBH were enumerated, while the rest were classified as 'shrubs'. Density of bamboo culms, shrubs, palms, bananas, and tall grass clumps was estimated in each of six 2 m radius circular plots laid at 5 m intervals on the transect, beginning from the starting point of the transect. Percentage cover of herbaceous forms and leaf litter, dead woody matter abundance and liana abundance in each circular plot were estimated visually. Percentage canopy cover from ground level was estimated with a hand-held canopy densiometer from the centre of each circular plot. Litter depth was gauged by pressing a blunt rod of 0.5-cm diameter at 5 random points in each circular plot, and counting the number of leaves pinned under it.

Principal Components Analysis (PCA) was used to identify different aspects of habitat change with vegetation succession and collapse the list of raw variables into composite factors that could potentially predict frog and lizard community structure. The factor structure was rotated using the Varimax method to obtain clear loading patterns [18]. As additional variables, within-habitat coefficient of variation (CV) of variables was also used along with the raw data as measures of habitat heterogeneity. Within habitat variation was considered potentially informative as it is an important feature of the adaptive landscape [30]. Habitat data were collected at comparable times of each month for all plots, and these CVs are unlikely to be due to temporal fluctuations. Data were square root transformed if they deviated from normality. For a list of variables used in the analyses, see Additional file 3.

As the objective of the analysis was to combine variables into composite, orthogonal factors that could potentially account for community and guild structure, all factors with eigenvalues = 0.8 were extracted, irrespective of the number of factors thus extracted. Although somewhat arbitrary, in essence this eigenvalue threshold ensured that a factor was included only if it extracted approximately as much as one raw variable [18]. Although all extracted factors were used as predictors of community structure (see below), in order to obtain a graphic, low dimensional representation of the two gradients of habitat recovery, scores of only the first two factors were plotted. Deterministic sets of variables that changed directionally with chronosere age were identified by regressing scores of each factor against chronosere ages.

### Frog and lizard sampling

The low abundance of amphibians and reptiles and unstandardised sampling methodology in tropical Asia reduces the reliability of species diversity estimates and hence community structure analyses [8]. Taking this problem into consideration, three techniques were used in conjunction to maximize inventorying effort – (i) belt transects, (ii) pitfall trapping and (iii) systematic searching. All these techniques are oriented towards sampling terrestrial, and non-canopy arboreal species, and to further increase the sampling efficiency, the study was restricted to terrestrial, non-fossorial, and non-canopy frogs (Amphibia: Anura) and lizards (Reptilia: Sauria, excluding family Varanidae). Although this excluded a few amphibian and reptile groups, it ensured that taxonomic groups unsuited for the chosen sampling techniques were not unnecessarily included, thus augmenting the reliability of the data. To distribute sampling effort effectively among the ten plots, sampling was carried out in sampling 'sessions' of ten days each. Eleven such sessions (= 110 days) were completed, starting from 15th December 1998, to the end of April 1999. Sufficient time was allocated to all three sampling techniques during each session.

#### Belt transects

To improve detection and gather information for delineation of EGs (see below), the traditional transect method was modified by eliminating pseudoreplication and sampling both nocturnal and diurnal species on the same transect [34,35]. The former was achieved by establishing fresh 50(length) × 3(width) × 3(height) m transects during each session, which were sampled only once. To detect both nocturnal and diurnal taxa, each transect was walked in both directions (to and fro) by two observers. The diurnal animal walk (DAW) was first, and was conducted at a steady pace fixed for all plots (ca. 20 min/50 m). Any animal seen leaving the transect area was recorded as being present on it. Care was taken to cause minimal disturbance to the habitat, and no active searches were done. The nocturnal animal walk (NAW), conducted in the direction opposite to the DAW, was focused on intensive microhabitat searching within the same 50 × 3 × 3 m area. All nocturnal animals found on the DAW were included in the analyses, but to reduce the possibility of re-recording the same animal, diurnal animals found on the NAW were not. Behavioural and microhabitat data were recorded for every animal detected (see below). All transects were sampled immediately after they were established, between 1000–1400 hrs during winter (mid-December to February) and 0900–1300 during summer (March and April). There was no noticeable species turnover with season, so winter and summer data were not analyzed separately [17]. The belt transects also yielded abundance data, which are not used in this paper [17].

Although time taken for the NAW was more or less constant *within *a chronosere, it varied considerably across plots. The DAW time on the other hand, was more or less consistent. This strategy was used because just as sampling effort needs to be proportional to habitat heterogeneity, higher microhabitat complexity calls for proportionally greater searching effort. Figure 7 shows how well this sampling strategy was implemented. There is a strong positive correlation between an index of microhabitat complexity (calculated as the sum of the coefficients of variance for various understorey habitat structure variables listed in Additional file 3) and time spent on the NAW, but not the DAW. Thus, though no extra time was needed to sight active (diurnal) animals in more complex habitats, the time needed for microhabitat searching (and hence NAW time) increased along a gradient of increasing habitat complexity from the 1-yr fallows and teak plots to mature forest. A total of 192 belt transects were completed, from a minimum of thirteen in jh1B to thirty-eight in jh35 (See Figure 7 for sample sizes).

#### Pitfall trapping

This technique was used to supplement species inventorying from the belt transects, and for an unbiased measure of the effects of weather on herpetofaunal activity and hence sampling efficiency. Comparisons of trapping frequency across plots over the study period are not used in this paper. Each pitfall array was, 'Y'-shaped, with three terminal (30 cm diam. × 60 cm depth) and one central (50 cm diam. × 70 cm depth) cylindrical aluminium funnel pitfall traps buried in the ground. The traps were connected with three opaque plastic-sheet fences (the arms of the 'Y') 0.4 m high and 5 m long, held up by bamboo stakes. In all, 22 arrays were placed, with two in each plot except for the large Jh35, which had four. Arrays were at comparable distance from plot edges, and on similar slope. Systematic trapping was initiated ten days after trap were established. Traps were opened for 5–10 consecutive days, and checked according to habitat characteristics, taking into consideration the level of exposure trapped animals were likely to be subjected to; plots with open habitat, such as jh1A were checked most (every alternate day) and those with relatively closed habitat such as matA were checked least frequently (every third day). Most specimens (95.2 %) obtained from pitfall trapping were released a minimum of 100 m away from the array, either in the same site, or in similar habitat elsewhere. A few were retained as voucher specimens.

#### Systematic searching

At the end of a sampling session in a plot, far ranging searches were carried out. This augmented species inventorying, and provided information crucial for EG classification (see below). Periodically, nocturnal searches were also made to collect information about the refuge of diurnally active animals, and also to confirm the presence or absence of species in different chronoseres.

#### Identification of taxa

Irrespective of the sampling technique, animals detected were caught whenever possible, and identified in hand. All those that escaped were identified to a justifiable level or excluded from the analyses. A few individuals of taxonomically problematic species or taxa were preserved for later identification.

### Sampling efficiency

The effectiveness of sampling was evaluated by species accumulation curves (see Additional file 4), and the effort adjusted after a mid-fieldwork examination of species richness data across chronoseres. While all the early succession stages and teak plantations reach an asymptote very soon, the 30–35 year fallow stopped yielding new species only by the eighth sampling session, while mature forest continued to yield new species till the final sampling session.

### Characterization of frog and lizard community succession

Overlap between recovering frog and lizard communities was measured with the Bray-Curtis measure between all possible pairs of chronoseres using presence absence data of all species (see Additional file 4). The resultant dissimilarity matrix was then used to generate a dendrogram using the UPGMA clustering algorithm [18]. Species turnover in sequential frog vs. lizard communities was compared using the mean Jaccard's dissimilarity value between all chronosere pairs calculated from separate presence absence data for the two groups [36].

### Ecological group classification and phylogenetic structure

Life history and behavioural traits were used to group species. These are often called guilds (e.g., [37]), but are referred to as ecological groups (EGs) here because the classification covers species from all chronoseres, including those that belonged to separate, non-interacting communities. The representatives of each EG in a particular community or chronosere on the other hand, can be considered a guild of that habitat's community. The life history and behavioural traits used for the EG classification were: diel activity period, habitat use when active, habitat use when resting, substrate temperature when active, air temperature when active, relative humidity when active, substrate moisture when active, resting refuge, resting refuge temperature, resting refuge substrate moisture and foraging tactic. To validate this data, information from literature and consultations with regional herpetologists was also used. These data, collected at different measurement scales, were rescaled to discrete categories to which species were allocated as absent or present. From this binary data, a dissimilarity matrix was calculated between all species using the Bray-Curtis measure [19]. The dissimilarity matrix was then scaled using non-metric multidimensional scaling (NMDS). NMDS geometrically represents dissimilarities in a graphical, low-dimensional space, and is a robust method to represent ecological distance [18,19]. See Additional file 4 for the list of species included in the EG classification.

Phylogenetic structure was measured as the ratio of number of species to the number of genera (S/G ratio) in each EG. A similar approach has been used in studies addressing questions about phylogenetic structure in ecological communities [15].

### Community – Habitat interrelationships

To test which habitat attributes influenced community structure, Mantel tests of correspondence between dissimilarity (distance) matrices [18,19,38,39] were used. Dissimilarity matrices between sites were generated based on differences in set of composite variables (factors) extracted by the PCA analysis (unsquared Euclidean distances), and for different levels of frog and lizard community composition (from entire community to guilds defined by the EG classification using Jaccard's index) [18,19]. Significance of correlation coefficients was tested by 1000 row-column Monte Carlo randomizations for each pair of matrices.

To test whether the availability of composite variables (PCA factors) that predicted community and guild structure identified by the matrix correspondence tests did indeed influence community succession and phylogenetic structure along gradients of habitat recovery, correlations between sums of factor scores and species richness, ratio of species number/guild number and S/G ratios across chronoseres were tested.

## Authors' contributions

SSP conceived the study, carried out the fieldwork, performed the data analyses, and drafted the manuscript. BCC and GSR participated in design and coordination of the study. GSR also supervised the vegetation identification and habitat classification. All authors read and approved the final manuscript.

## Supplementary Material

Additional File 2 **Photographs of habitat types. **Representative photographs of habitat types Click here for file

Additional File 3 **Results of PCA along with list of habitat variables used. **PCA Results Click here for file

Additional File 4 **Species accumulation curves and frog and lizard species' lists. **Species accumulation curves and lists Click here for file

Additional File 1 **Maps of study area. **Location map of study area sampling plots with respect to vegetation types Click here for file
