# Supplementary material for: Recovery of frog and lizard communities following primary habitat alteration in Mizoram, Northeast India
Source: BMC Ecol. 2004 Aug 6;4:10. doi: 10.1186/1472-6785-4-10 (PMC514559; doi:10.1186/1472-6785-4-10)
Supplement: Additional File 2 — Photographs of habitat types. Representative photographs of habitat types [file 1472-6785-4-10-S2.pdf]

Representative photographs of chronoseres

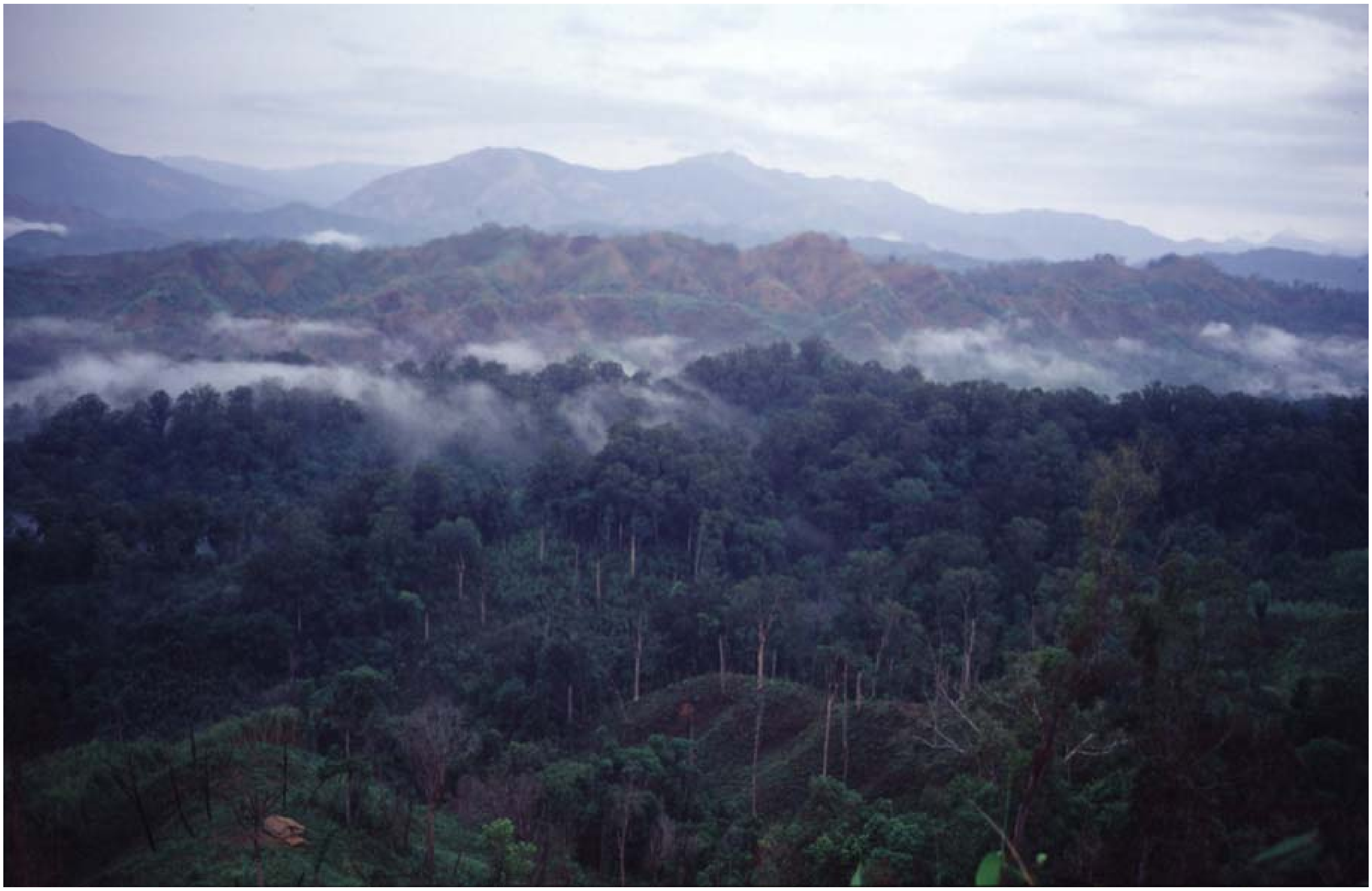

A part of the study area outside the southern limits of Ngengpui Wildlife Sanctuary. Note the habitat mosaic, with early post-jhum successional habitats in the foreground. The hut seen in the foreground is used by the cultivator for the period of the *jhum* cultivation (one year in the case of this area).

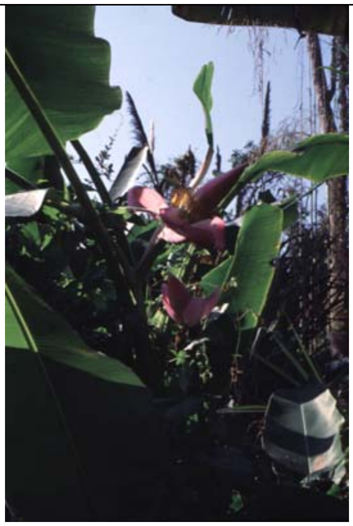 1-yr *jhum* fallow. Note the profuse growth of herbaceous plants.	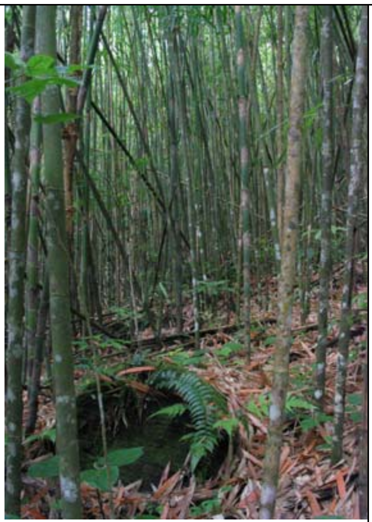 10-yr *jhum* fallow. Note the almost uniform distribution of the non-sympodial (non-clump forming) bamboo *Melocanna baccifera*.	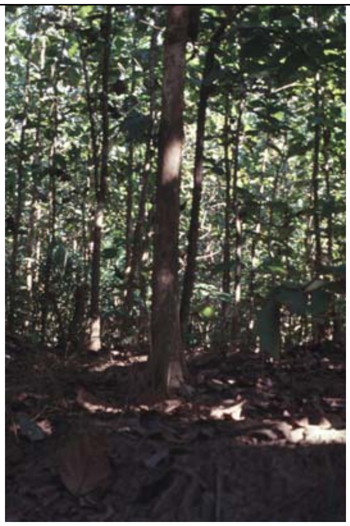 22 year teak (*Tectona grandis*) plantation. Note the lack of shrub cover in the understory, and the coarse, large-leaved teak leaf litter.	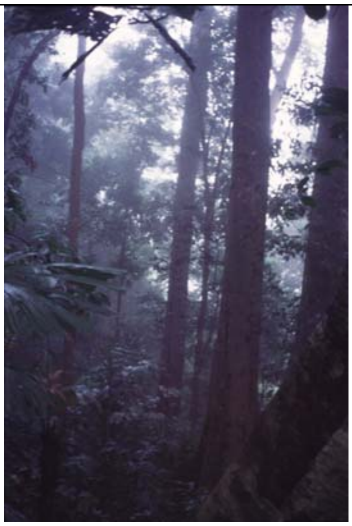 Mature forest. Note the complex habitat structure.
