# Supplementary material for: Recovery of frog and lizard communities following primary habitat alteration in Mizoram, Northeast India
Source: BMC Ecol. 2004 Aug 6;4:10. doi: 10.1186/1472-6785-4-10 (PMC514559; doi:10.1186/1472-6785-4-10)
Supplement: Additional File 3 — Results of PCA along with list of habitat variables used. PCA Results [file 1472-6785-4-10-S3.pdf]

PCA Results:

	Eigenvalue	% total Variance	Cumulative Eigenvalues	Cumulaltive %
**Factor1**	13.3213	40.36758	13.3213	40.36758
**Factor2**	6.264356	18.9829	19.58566	59.35047
**Factor3**	2.232367	6.76475	21.81802	66.11522
**Factor4**	1.721152	5.215612	23.53918	71.33083
**Factor5**	1.611187	4.882386	25.15036	76.21322
**Factor6**	1.250278	3.788721	26.40064	80.00194
**Factor7**	1.133505	3.434865	27.53415	83.43681
**Factor8**	0.79116	2.397454	28.32531	85.83426

Factor Loadings (Varimax normalized)

Variable loadings >0.65 are marked with **blue**, and >0.50 with **red**.

Variable	Factor 1	Factor 2	Factor 3	Factor 4	Factor 5	Factor 6	Factor 7	Factor 8
Bamboo density	**0.79**	-0.49	-0.01	0.02	-0.09	0.16	0.19	0.01
Banana plant density	**-0.54**	-0.29	0.06	0.29	**0.56**	-0.17	-0.24	0.04
Canopy cover	**0.70**	**0.53**	0.23	-0.15	-0.25	0.02	0.07	-0.16
Canopy Height	0.34	**0.81**	0.16	-0.14	-0.22	0.04	0.09	-0.11
CV of Canopy Cover	**-0.74**	-0.44	-0.30	0.12	-0.05	0.05	0.07	0.19
CV of Canopy Height	**-0.55**	-0.03	-0.22	**0.55**	0.20	0.06	0.19	-0.04
CV of GBH	0.02	**0.80**	0.21	-0.10	-0.28	0.14	-0.15	-0.07
CV of herb cover	0.13	0.30	**0.88**	-0.01	-0.10	0.03	0.00	-0.01
CV of herb height	0.14	0.24	**0.91**	0.00	-0.05	0.04	0.10	0.00
CV of litter cover	**-0.80**	-0.36	-0.27	0.03	0.05	0.05	-0.09	0.23
CV of Litter Depth	**-0.73**	-0.17	-0.10	-0.24	0.37	0.06	0.18	0.18
CV of organic matter content	**-0.59**	-0.28	-0.34	0.22	0.01	0.17	-0.12	0.40
CV of shrub density	0.03	-0.10	0.17	-0.04	0.05	**0.91**	-0.07	-0.15
CV soil moisture	-0.18	0.02	0.04	0.04	0.32	-0.33	-0.06	**0.71**
Diversity of leaf litter	-0.09	0.17	-0.26	-0.08	**0.81**	0.09	-0.20	0.15
Herb (ground) cover	-0.44	-0.19	**-0.64**	0.38	0.03	0.02	-0.01	0.23
Herb height (average)	-0.24	-0.21	**-0.87**	-0.05	0.19	-0.14	-0.04	-0.08
Liana abundance	0.14	**0.87**	0.19	0.18	0.06	-0.09	0.03	0.07
Liana girth diversity	0.16	**0.89**	0.18	0.21	0.03	-0.10	-0.02	0.03
Litter (ground) cover	**0.80**	0.36	0.31	-0.13	-0.04	-0.01	0.13	-0.08
Litter depth	**0.77**	-0.12	0.12	-0.10	0.06	0.03	0.35	0.30
Shrub size diversity	0.26	**0.77**	0.11	0.10	0.15	0.00	0.07	-0.22
Shrubs density	-0.10	0.41	0.14	**0.57**	-0.17	-0.30	-0.10	0.19
Soil moisture	0.22	0.18	0.12	0.04	-0.20	-0.08	**0.85**	-0.06
Soil organic matter content	**0.68**	**0.56**	0.13	-0.07	0.06	-0.08	0.24	-0.17
Tall grass density	**-0.65**	-0.44	-0.16	-0.05	0.41	-0.11	-0.15	0.06
Tree density	0.03	**0.71**	0.12	**-0.58**	-0.08	-0.12	-0.08	0.08
Tree girth at breast height (GBH)	0.03	**0.86**	0.08	-0.20	-0.02	-0.01	0.18	0.10
Tree species richness	0.24	**0.88**	0.18	0.13	0.07	-0.05	0.05	0.02
Understory palm abundance	-0.02	**0.84**	0.37	0.15	0.01	-0.06	0.12	-0.05
Woody matter abundance-bamboo	**0.82**	-0.40	-0.06	0.05	-0.03	0.23	-0.05	0.14
Woody matter abundance-logs	**-0.59**	0.15	-0.17	0.24	**0.53**	0.14	0.16	0.27
Woody matter abundance-twigs	-0.24	**0.81**	0.00	-0.27	0.11	-0.07	0.03	-0.05
Explained Variance	7.608	9.16	3.827	1.731	2.063	1.323	1.335	1.279
Proportion of total	0.231	0.278	0.116	0.052	0.063	0.04	0.04	0.039

Factor scores

Plot	Factor 1	Factor 2	Factor 3	Factor 4	Factor 5	Factor 6	Factor 7	Factor 8
**jh1A**	-1.548	-0.901	-0.464	0.864	0.87	0.008	0.047	0.805
**jh1B**	-1.467	-1.061	-0.624	1.358	0.526	-0.065	-0.135	-0.112
**jh5**	0.938	-1.196	0.241	-0.193	-0.368	0.278	1.307	-0.159
**jh10**	1.102	-0.998	0.605	0.153	-0.194	0.131	-0.518	-0.045
**tk4**	-0.734	-0.53	0.225	-2.448	0.617	-0.633	-0.865	-0.337
**tk22**	-1.092	-0.114	-0.809	-1.724	-1.782	0.256	-0.63	-0.192
**jh35**	1.194	0.599	-1.284	0.122	0.467	0.455	-0.535	0.522
**matA**	-0.364	1.085	0.834	0.042	-0.082	-0.146	0.605	0.071
**matB**	-0.104	1.266	0.809	0.271	0.044	0.018	-0.064	-0.06
**matC**	0.056	1.031	-0.339	0.485	-0.267	-0.695	-0.021	-0.818
