# Supplementary material for: Recovery of frog and lizard communities following primary habitat alteration in Mizoram, Northeast India
Source: BMC Ecol. 2004 Aug 6;4:10. doi: 10.1186/1472-6785-4-10 (PMC514559; doi:10.1186/1472-6785-4-10)
Supplement: Additional File 4 — Species accumulation curves and frog and lizard species' lists. Species accumulation curves and lists [file 1472-6785-4-10-S4.pdf]

Species accumulation curves and species lists:

Species accumulation across for frogs and lizards across habitats. (A) 1 yr. *Jhum* fallows (B) 4-10 yr. *Jhum* fallows (C) Teak plantations (D) 30-35 yr. *Jhum* fallow (E) Mature forest.

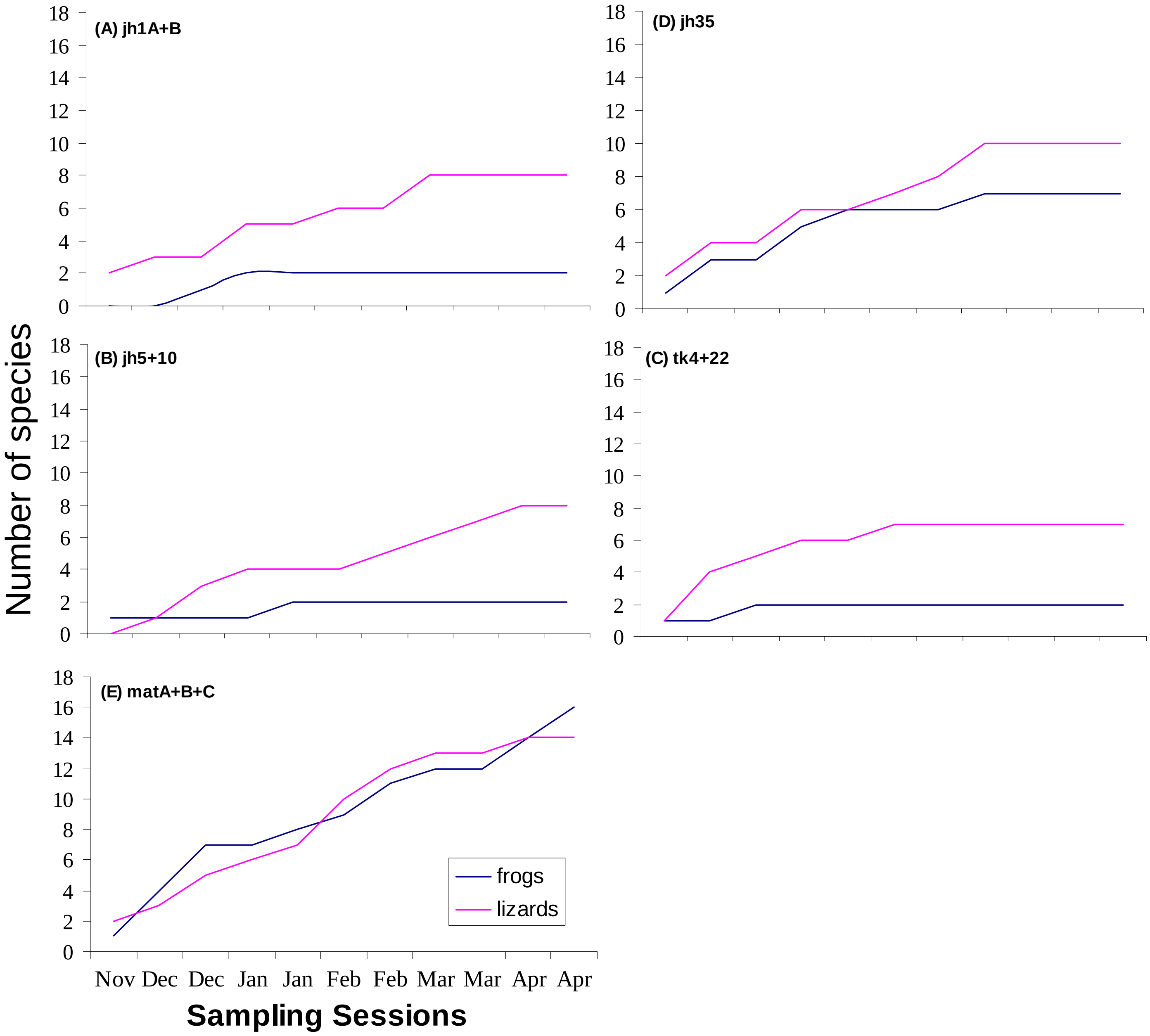

List of species across all habitats, with ecological group (EG) classification

Species	EG	Plots (Present = 1; Absent = 0)									
**Frogs:**		**jh1A**	**jh1B**	**jh5**	**jh10**	**tk4**	**tk22**	**jh35**	**matA**	**matB**	**matC**
Pterorana khare	**-**	0	0	0	0	0	0	1	0	0	1
Microhyla berdmorei	**CT**	0	0	0	0	0	0	0	1	1	0
Xenophrys parva	**CT**	0	0	0	0	0	0	1	1	0	0
Leptobrachium smithi	**CT**	0	0	0	0	0	0	1	0	1	0
Bufo melanostictus	**CT**	1	0	0	0	1	1	0	0	1	0
Phrynoglossus cf. tenasserimensis	**CT**	0	0	0	0	0	0	1	1	1	1
Rana alticola	**CT**	0	0	0	0	0	0	1	1	1	1
Rana laticeps	**CT**	0	0	0	1	0	0	1	0	0	1
Kaloula pulchra	**NA**	0	0	0	0	0	0	0	1	1	1
Pedostibes kempi	**NA**	0	0	0	0	0	0	0	1	1	0
Philautus parvulus	**NA**	0	1	0	1	0	1	1	1	1	1
Philautus sp.(1)	**NA**	0	0	0	0	0	0	0	0	1	1
Philautus sp.(2)	**NA**	0	0	0	0	0	0	1	1	1	1
Philautus namdaphaensis	**NA**	0	0	0	0	0	0	0	0	1	0
Chirixalus vittatus	**NA**	0	0	0	0	0	0	0	1	1	0
Rhacophorus maximus	**NA**	0	0	0	0	0	0	0	0	0	1
**Total frogs**		**1**	**1**	**0**	**2**	**1**	**2**	**8**	**9**	**12**	**9**
**Lizards:**											
Cosymbotus platyurus	**NA**	1	0	0	0	0	0	1	1	1	0
Ptychozoon lionotum	**NA**	0	0	0	0	0	0	0	0	0	1
Gekko gecko	**NA**	0	0	0	0	0	0	1	1	1	1
Hemidactylus garnoti	**NA**	1	1	0	1	0	0	1	1	0	0
Draco maculatus	**DA**	0	0	0	0	0	0	0	1	1	1
Calotes versicolor	**DA**	1	1	1	1	1	1	0	0	0	0
Calotes emma	**DA**	0	0	0	1	0	0	1	1	1	1
Calotes cf. alticristatus	**DA**	0	0	1	1	0	0	1	0	0	1
Ptyctolaemus gularis	**DA**	0	0	0	0	0	0	1	0	1	1
Takydromus sexlineatus	**DT**	0	1	1	1	1	1	1	0	1	0
Mabuya multifasciata	**DT**	1	1	1	1	1	1	0	0	0	0
Mabuya macularia	**DT**	1	1	1	1	1	1	0	0	1	0
Mabuya sp.	**DT**	1	0	1	1	1	0	1	1	0	1
Sphenomorphus indicus	**DT**	0	0	0	0	0	0	1	1	1	1
Sphenomorphus maculatum	**DT**	1	1	1	1	1	1	0	1	0	1
Sphenomorphus courcyanum	**DT**	0	0	0	0	0	0	1	0	1	1
Tropidophorus assamensis	**CT**	0	0	0	0	0	0	1	0	1	1
**Total lizards**		**7**	**6**	**7**	**9**	**6**	**5**	**11**	**8**	**10**	**11**
**POOLED TOTAL**		**8**	**7**	**7**	**11**	**7**	**7**	**19**	**17**	**22**	**20**
