# Supplementary material for: Recovery of frog and lizard communities following primary habitat alteration in Mizoram, Northeast India
Source: BMC Ecol. 2004 Aug 6;4:10. doi: 10.1186/1472-6785-4-10 (PMC514559; doi:10.1186/1472-6785-4-10)
Supplement: Additional File 1 — Maps of study area. Location map of study area sampling plots with respect to vegetation types [file 1472-6785-4-10-S1.pdf]

Location map of study area sampling plots with respect to vegetation types

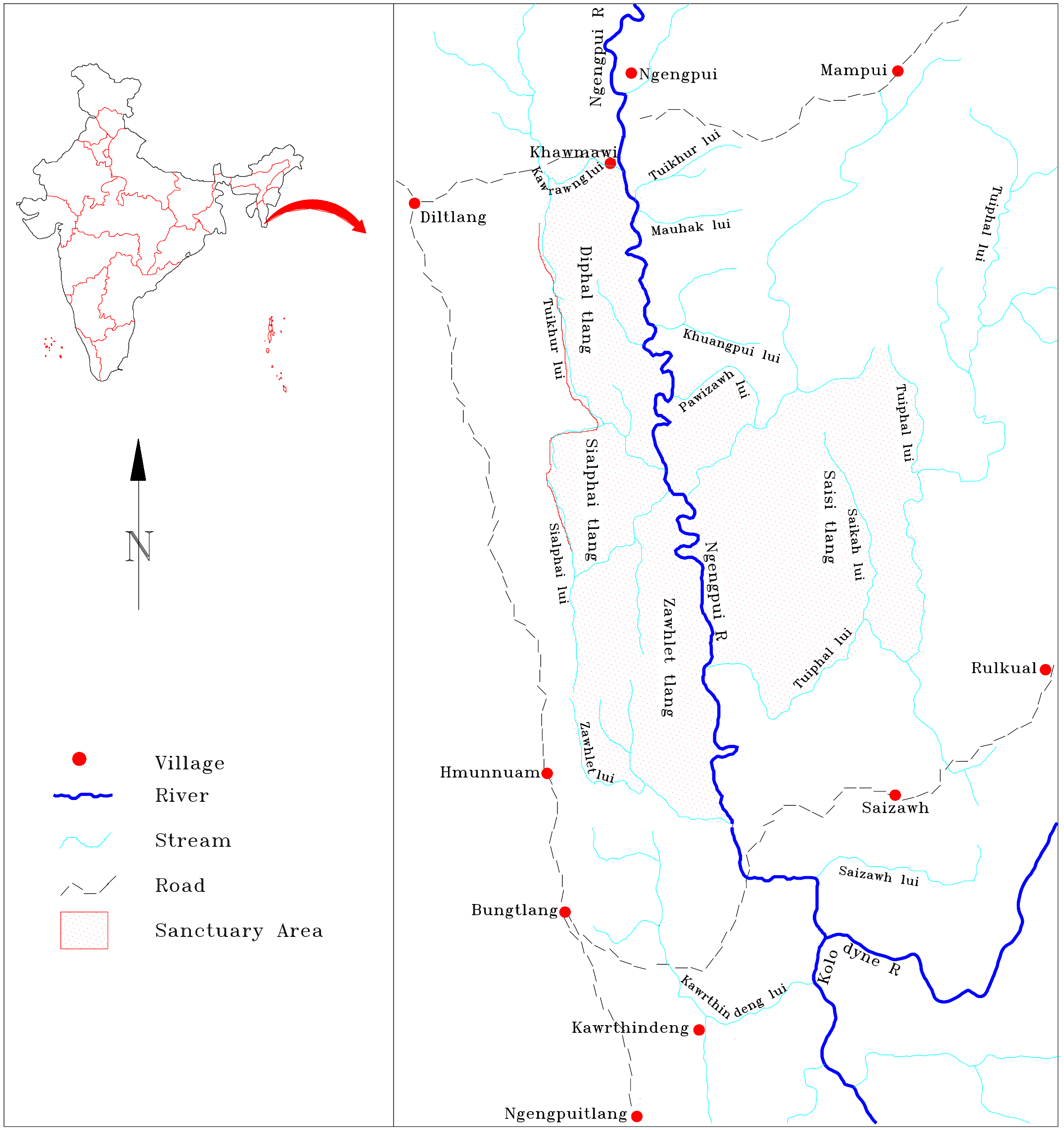

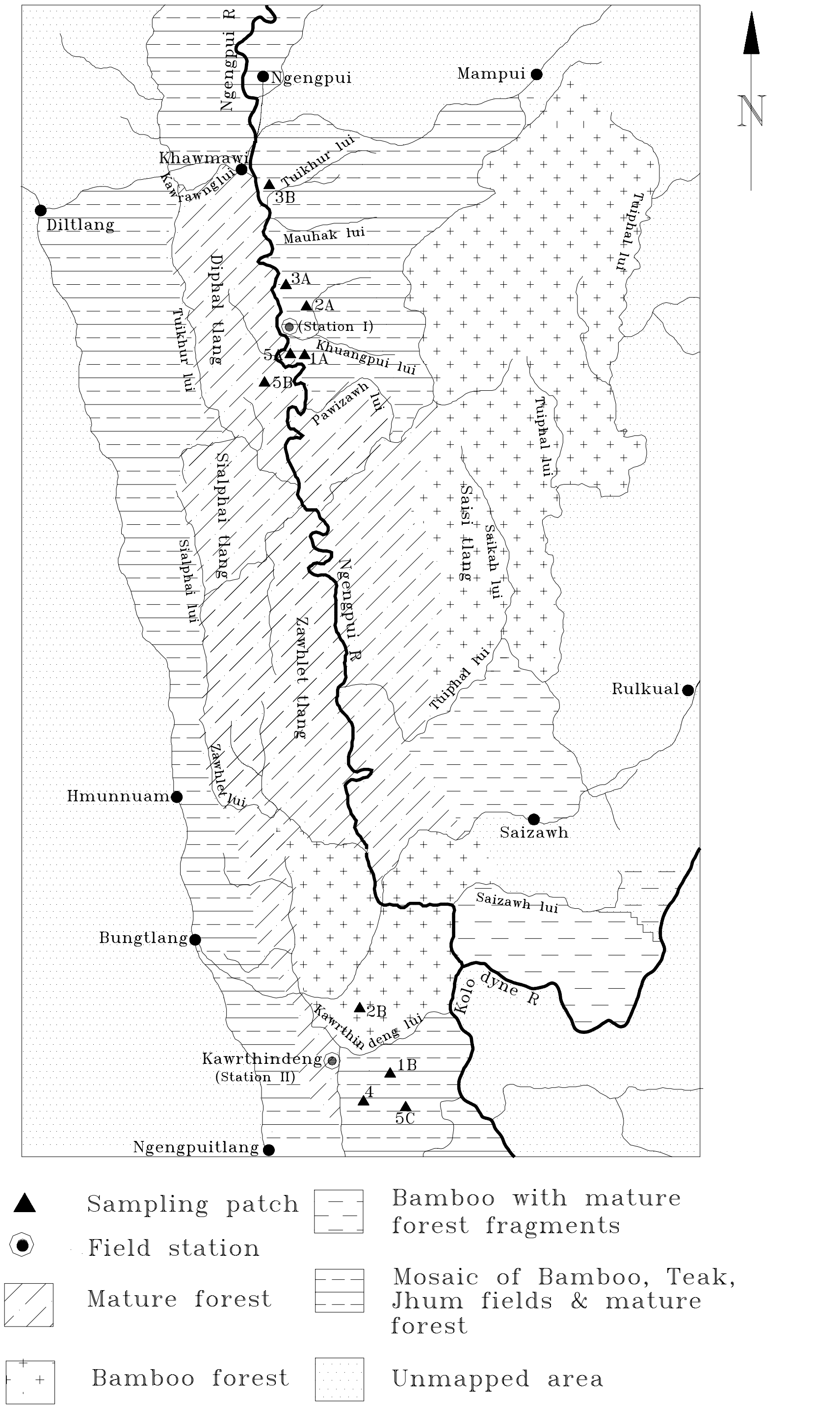

Sampling plots-

1A& B : 1yr *jhum* fallows

4 : 30-35yr. *jhum* fallows

2A & 2B : 4-10yr fallows

5A,B&C : Mature forest

3A & 3B : teak plantations
